# X-ray free electron laser observation of ultrafast lattice behaviour under femtosecond laser-driven shock compression in iron

**DOI:** 10.1038/s41598-023-40283-6

**Published:** 2023-08-31

**Authors:** Tomokazu Sano, Tomoki Matsuda, Akio Hirose, Mitsuru Ohata, Tomoyuki Terai, Tomoyuki Kakeshita, Yuichi Inubushi, Takahiro Sato, Kohei Miyanishi, Makina Yabashi, Tadashi Togashi, Kensuke Tono, Osami Sakata, Yoshinori Tange, Kazuto Arakawa, Yusuke Ito, Takuo Okuchi, Tomoko Sato, Toshimori Sekine, Tsutomu Mashimo, Nobuhiko Nakanii, Yusuke Seto, Masaya Shigeta, Takahisa Shobu, Yuji Sano, Tomonao Hosokai, Takeshi Matsuoka, Toshinori Yabuuchi, Kazuo A. Tanaka, Norimasa Ozaki, Ryosuke Kodama

**Affiliations:** 1 Graduate School of Engineering, Osaka University, 2-1 Yamada-Oka, Suita, Osaka 565-0871 Japan; 2 SANKEN, Osaka University, Ibaraki, Osaka 567-0047 Japan; 3 Fukui University of Technology, Fukui, 910-8505 Japan; 4 Japan Synchrotron Radiation Research Institute, 1-1-1 Kouto, Sayo, Hyogo 679-5198 Japan; 5 RIKEN, SPring-8 Center, 1-1-1 Kouto, Sayo, Hyogo 679-5148 Japan; 6 SLAC National Accelerator Laboratory, Stanford, CA 94309 USA; 7 Next Generation TATARA Co-Creation Centre, Shimane University, Matsue, Shimane 690-8504 Japan; 8 Graduate School of Engineering, The University of Tokyo, Tokyo, 113-8656 Japan; 9 Institute for Integrated Radiation and Nuclear Science, Kyoto University, Kumatori, Osaka 590-0458 Japan; 10 Graduate School of Advanced Science and Engineering, Hiroshima University, Higashihiroshima, Hiroshima 739-8511 Japan; 11 Center for High Pressure Science and Technology Advanced Research, Shanghai, 201203 China; 12 Institute of Industrial Nanomaterials, Kumamoto University, Kumamoto, 860-8555 Japan; 13 Kansai Institute for Photon Science (KPSI), National Institutes for Quantum Science and Technology (QST), Kizugawa, Kyoto 619-0215 Japan; 14 Graduate School of Science, Osaka Metropolitan University, Osaka, 558-8585 Japan; 15 Graduate School of Engineering, Tohoku University, Miyagi, 980-8579 Japan; 16 Sector of Nuclear Science Research, Japan Atomic Energy Agency, Sayo, Hyogo 679-5148 Japan; 17 Institute for Molecular Science, National Institutes of Natural Sciences, Okazaki, 444-8585 Japan; 18 Toshiba Energy Systems & Solutions Corporation, Kawasaki, Kanagawa 212-0013 Japan; 19 Institute for Open and Transdisciplinary Research Initiatives, Osaka University, Suita, Osaka 565-0871 Japan; 20 Institute of Laser Engineering, Osaka University, Suita, Osaka 565-0871 Japan

**Keywords:** Surfaces, interfaces and thin films, Mechanical properties

## Abstract

Over the past century, understanding the nature of shock compression of condensed matter has been a major topic. About 20 years ago, a femtosecond laser emerged as a new shock-driver. Unlike conventional shock waves, a femtosecond laser-driven shock wave creates unique microstructures in materials. Therefore, the properties of this shock wave may be different from those of conventional shock waves. However, the lattice behaviour under femtosecond laser-driven shock compression has never been elucidated. Here we report the ultrafast lattice behaviour in iron shocked by direct irradiation of a femtosecond laser pulse, diagnosed using X-ray free electron laser diffraction. We found that the initial compression state caused by the femtosecond laser-driven shock wave is the same as that caused by conventional shock waves. We also found, for the first time experimentally, the temporal deviation of peaks of stress and strain waves predicted theoretically. Furthermore, the existence of a plastic wave peak between the stress and strain wave peaks is a new finding that has not been predicted even theoretically. Our findings will open up new avenues for designing novel materials that combine strength and toughness in a trade-off relationship.

## Introduction

Highly compressed states via shock waves have been essential for understanding various phenomena such as material synthesis^1^ and strengthening^2^, high-velocity impacts^3^, planet formation^4^, and inertial confinement fusion^5^. Material properties such as mechanical, optical, electrical, and magnetic ones change drastically on an ultrashort timescale when subjected to shock compression^6,7^. These studies have mainly used explosives, plate impacts and high-power lasers as shock drivers, mainly because such shock drivers can transiently create a thermodynamically steady and thermally equilibrium shocked state, i.e. the Hugoniot state^8,9^ in the material.

The femtosecond laser is a relatively new shock-driven tool that has been in use for about 20 years^10–13^. Direct femtosecond laser irradiation of a thin aluminium film produces a shock pressure of 100 to 300 GPa, depending on the laser intensity, estimated under the assumption of the Hugoniot state^13^. The femtosecond laser-driven shock wave in metal causes plastic deformation and, if the material has high-pressure phases, high-pressure phase transition, leaving unique traces such as unique dislocation structures^14,15^ and the high-pressure phase of iron^16^ that cannot be obtained by conventional compression techniques. Furthermore, the plastic deformation induced by direct femtosecond laser irradiation of metals has been applied to strengthen the metals as a new laser peening technique without any sacrificial overlay under atmospheric conditions, called dry laser peening (DLP)^17,18^, whereas conventional laser peening techniques using nanosecond pulsed lasers require sacrificial overlays such as protective coatings and plasma confinement media^19–21^.

Characteristics of the femtosecond laser-driven shock wave, such as shock profile and peak pressure, have been diagnosed experimentally using ultrafast pump and probe schemes^10–13,22^ such as ultrafast interferometry and ultrafast dynamic ellipsometry. Existing studies, except for Evans’ study^13^, have used a plasma confinement scheme, i.e. the pump laser passes through the glass substrate and irradiates the thin metal film deposited on the glass substrate, and the probe laser irradiates the free surface of the film. Although this scheme drives a shock wave and its characteristics have been thoroughly studied^10–12^, there is a concern that electrons and ions ejected from the metal during the early stage of the femtosecond laser irradiation may affect the shock formation due to preheating or plasma expansion because the laser-irradiated metal surface is the interface with the glass substrate and the ejected electrons and ions are confined in the interface^23–26^. Evans et al. measured the ultrafast behaviour of the backside of the metal when the pump laser was irradiated to the free surface of the metal and reported it to be driven by a shock pressure of 100 to 300 GPa assuming the Hugoniot state^13^. However, it is unclear whether the shock wave driven by direct femtosecond laser irradiation is applicable in the Hugoniot state. Furthermore, ultrafast interferometric and spectroscopic techniques can provide information on the ultrafast behaviour of laser-driven waves from nanometric order displacements with picosecond temporal resolution^10–13,22^. However, they cannot provide direct information on the lattice level behaviour, which is critical for understanding the elasto-plastic and phase transition behaviour under shock compression^27–30^.

Time-resolved X-ray diffraction (XRD) combined with laser-driven shock has been widely used to observe fast lattice behaviour^31–34^. X-rays from laser-produced plasma have mostly been used to study lattice behaviour such as structural phase transitions. The laser pulse duration is typically sub-nanoseconds or longer to produce a high X-ray flux, resulting in insufficient temporal resolution to observe lattice behaviour at the picosecond or femtosecond resolution. The X-ray free electron laser (XFEL) has successfully produced brilliant femtosecond X-ray pulses^35,36^. By combining an XFEL with an optical laser pulse, it is possible to investigate shock phenomena at femtosecond resolution^30^. The XFEL has been used to investigate shock compression states produced by a laser pulse with a pulse duration of sub-nanoseconds or longer^30,37–39^. In this case, the initial part of the laser pulse generates the plasma, while the rest of the laser pulse keeps the plasma stable by inverse bremsstrahlung, which pushes the material to form a steady shock wave, thereby creating a Hugoniot state^20^. However, in the case of direct femtosecond laser irradiation, there is no interaction between the ablation plasma and the laser pulse, as ablation occurs after the entire laser pulse has been deposited in the material^40^. For this reason, simulations have predicted that the mechanism of shock wave formation is different from that of conventional shock waves^41^. Because the femtosecond laser-driven shock wave behaves differently from conventional shock waves, unique microstructures^14–16^ are expected to be formed in materials by shock waves driven by direct femtosecond laser irradiation. However, the lattice behaviour of metals under shock compression driven by this direct femtosecond laser irradiation has never been investigated and remains experimentally unresolved.

In this study, we used the XFEL at SACLA^36^ to investigate the ultrafast lattice behaviour in iron directly irradiated by a femtosecond laser pulse. Iron was chosen as a reference material to evaluate the properties of shock waves driven by direct femtosecond laser irradiation, because iron is an important material in industrial and geoscientific fields and its behaviour under conventional shock compression has been thoroughly studied^33,38,42–53^.

## Methods

Polycrystalline iron with a purity of 99.99% (Kojundo Chemical Laboratory Co., Ltd., 10 × 10 mm, 1 mm thickness) was used as the target material. It was annealed at 1123 K under a low pressure of 10^–2^–10^–3^ Pa for 1 h to remove the residual strain, and its surface was then mirror-finished using colloidal silica. The average grain size of the annealed iron was measured to be 63 µm using the electron backscatter diffraction method.

**Figure 1:**
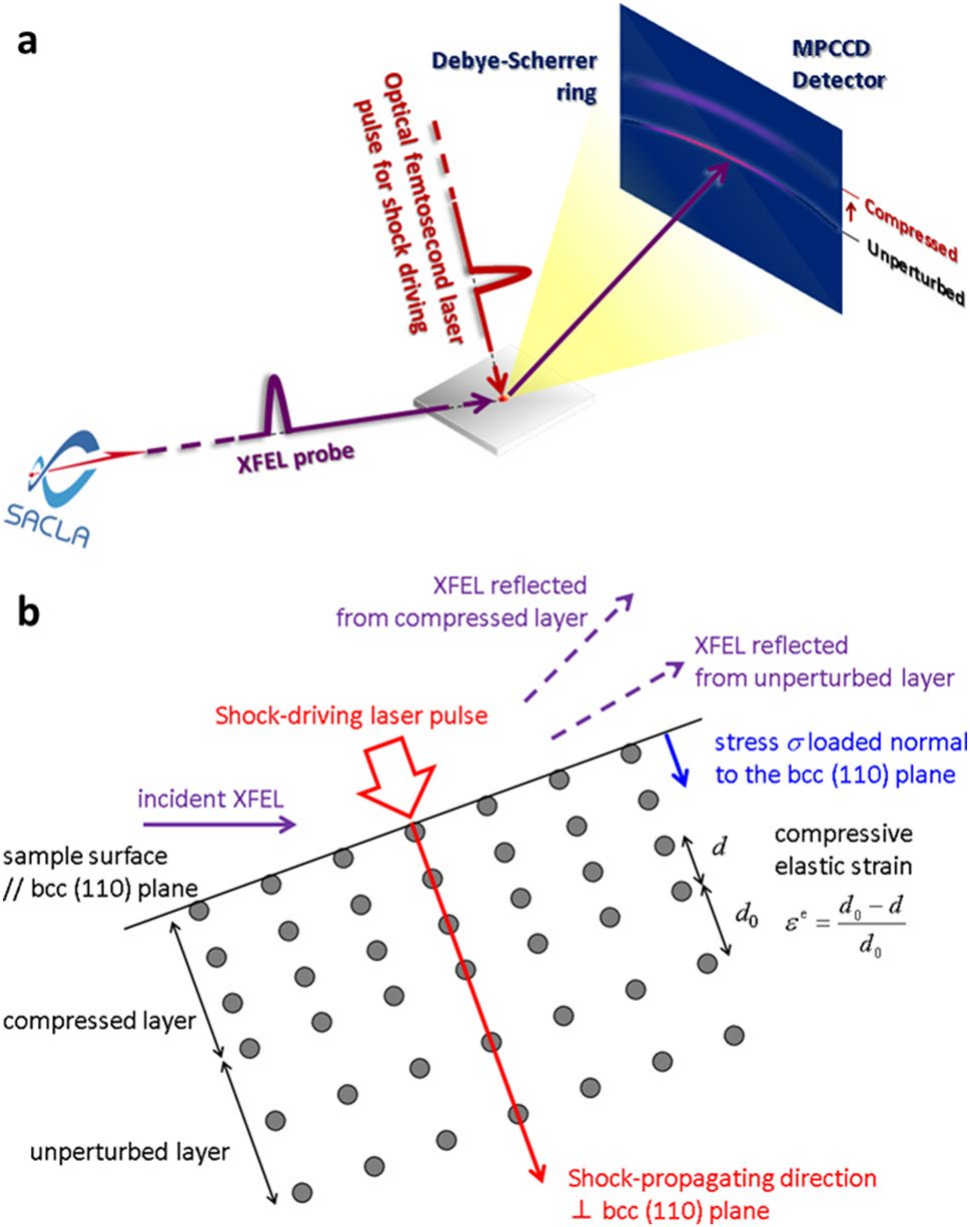
Schematic illustration of the optical femtosecond laser-pump and XFEL-probe experimental setup. (**a**) A pump-laser pulse is focused onto the 600 µm diameter spot on the iron surface. An XFEL pulse irradiates the sample maintaining a spatial overlap with the pump laser. Shot-to-shot XRD patterns are recorded with a two-dimensional MPCCD detector. (**b**) The sample surface is almost parallel to the bcc (110) plane of iron with a bcc structure. The direction of shock propagation is perpendicular to the bcc (110) plane, so stress *σ* is applied perpendicular to the plane. The diffracted X-ray beam from the compressed plane with the lattice spacing *d* is recorded at a higher angle than that from the unperturbed plane with the initial lattice spacing *d*_0_, where the compressive elastic strain *ε*^e^ is expressed by *ε*^e^ = (*d*_0_ – *d*)/*d*_0_.

The pump-probe experiment was performed in the experimental hatch EH3 at the beamline BL3 of SACLA^35^. Figure 1 shows a schematic illustration of the experimental setup. Pump laser-pulses with a wavelength of 800 nm, pulse duration of 43 fs, pulse energy of 60 mJ, and contrast ratio of 10^–6^ (Coherent Inc., Hidra-100) irradiated the target surface with a spot diameter of 600 µm, corresponding to an average intensity of 4.9 × 10^14^ W/cm^2^, which is almost the same as the intensity for femtosecond laser-shock processing treatment^14–18^. The shock front propagates into the material normal to the surface. XFEL pulses with a duration of 10 fs and a photon energy of 10 keV with an energy spread of 5 × 10^–3^ irradiated the sample while maintaining spatial overlap with the pump laser. The glancing angle between the XFEL beam and the target surface was 20°, while the pump laser irradiated the target with a normal incidence. The XFEL beam was cut with a slit of 70 μm (vertical) × 300 μm (horizontal). The XFEL irradiation area over the sample surface is therefore 204 μm × 300 μm, which fits within the *ϕ* 600 μm pump laser irradiation area. Spatial overlap between pump and probe lasers was ensured by using a Ce:YAG, which fluoresce to both pump and probe lasers. The timing 0 was adjusted using optical delay line of the femtosecond laser so that the timing of the fluorescence generated by XFEL irradiation of Ce:YAG was located at the rise of the fluorescence generated by femtosecond laser irradiation of Ce:YAG. The XFEL and pump-laser pulses were synchronised in time with shot-to-shot fluctuation of sub-picosecond. The delay time *τ* of the XFEL pulse from the femtosecond laser pulse was varied using an optical delay line along the optical laser path. The probe depth of the 10 keV X-ray from the surface was 1.12 μm. A two-dimensional multi-port charge-coupled device (MPCCD) detector was positioned so that the normal line from the centre crossed the spot of the pump laser pulse on the target. Shot-to-shot XRD patterns were recorded using the MPCCD. The angle between the incident XFEL and the normal line of the detector was 36°. The distance between the spot and the detector, calibrated using a gold target, was 138.02 mm.

Transmission electron microscopy (TEM: JEM-2010; JEOL) with an accelerating voltage of 200 kV was performed on femtosecond laser-driven shocked iron to observe its microstructure and lattice defects such as dislocations. To perform TEM, a small piece of the cross-section was extracted from the iron surface, and its thickness was reduced using a 30 keV focused Ga+ ion beam (FB-2000A; HITACHI). The sample surface was covered with tungsten before fabrication to prevent damage from ion beam bombardment. The dislocation density was estimated quantitatively using Ham's equation *ρ* = 2*N*/*Lt*, where *ρ* is the dislocation density, *N* is the number of intersections between dislocation lines and grid lines drawn on the TEM micrograph, *L* is the total length of the grid lines, and *t* is the thickness of the TEM sample^54^.

## Results

**Figure 2:**
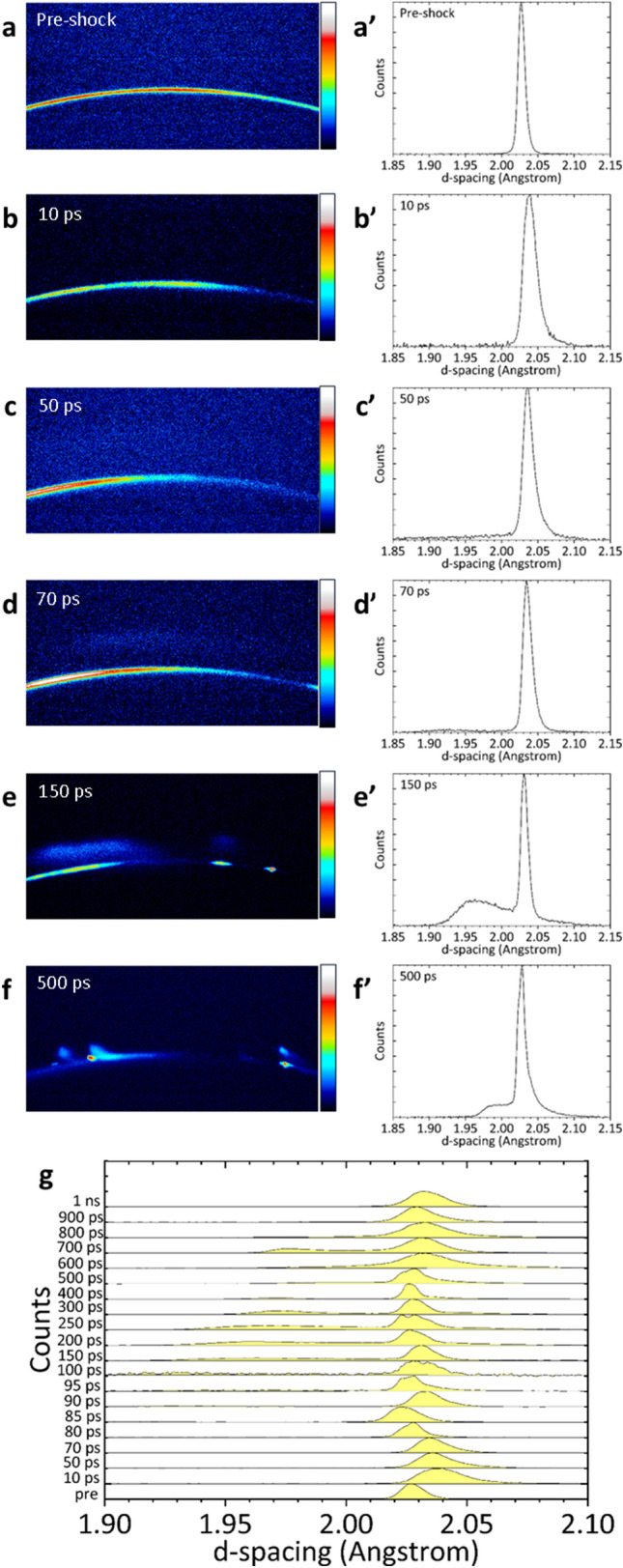
Diffraction patterns recorded at different delay times *τ* and the corresponding one-dimensional profiles. (**a**) The unperturbed pattern recorded before pump laser irradiation, pre-shocked state. A clear Debye–Scherrer ring indicates the Bragg peak for the (110) plane of iron with a bcc structure at 35.62°. (**b**) At *τ* = 10 ps, the ring becomes slightly wider. (**c**) At *τ* = 50 ps, an increase in baseline intensity in the higher angle region is observed relative to the initial Bragg peak. (**d**) At *τ* = 70 ps, a prominent new peak appears in the higher angle region. (**e**) At *τ* = 150 ps, the intensity of the new peak increases. (**f**) At *τ* = 500 ps, the peak shifts to a lower angle. This new peak indicates the Bragg one for the compressed bcc (110) plane. (**a′**–**f′**) are one-dimensional profiles corresponding to (**a**–**f**) where the XRD patterns obtained on the two-dimensional detector are intensity-integrated in the *δ* direction, 2*θ* is converted to d-spacing using *λ* = 2*d*sin*θ* relationships, where *λ* is the wavelength of the XFEL, *d* is a d-spacing of a lattice, *θ* is the Bragg angle, and the intensity is normalised to the maximum value. (**g**) One-dimensional profiles for a series of delay times.

Figure 2a to f show the typical diffraction patterns recorded at different delay times. The vertical direction in the figure represents the angle in the 2*θ* direction, and the circumferential direction of the diffraction ring represents the angle in the azimuthal *δ* direction. Figure 2a shows the unperturbed pattern recorded before pump-laser irradiation. A clear Debye–Scherrer ring was observed, indicating the Bragg peak for the (110) plane of iron with a body-centred-cubic (bcc) structure at 35.62°. The ring broadened slightly at *τ* = 10 ps (Fig. 2b). In Fig. 2c, the profile of this ring remained the same even at *τ* = 50 ps. However, an increased baseline intensity was observed in the higher-angle region of the initial Bragg peak, which became a distinct peak after *τ* = 70 ps (Fig. 2d to f). Subsequently, the peak shifts to a lower diffraction angle as the delay time increases.

One dimensional profiles at each delay time are shown in Fig. 2a′ to f′, corresponding to the two-dimensional patterns shown in Fig. 2a to f, where the XRD patterns obtained on the two-dimensional detector are intensity-integrated in the *δ* direction and 2*θ* is converted to d-spacing using the *λ* = 2*d*sin*θ* relationship, where *λ* is the wavelength of the XFEL, *d* is a d-spacing of the lattice, and *θ* is the Bragg angle. Furthermore, the intensity is normalised to the maximum value. The d-spacing of the (110) plane increases slightly at *τ* = 10 ps. The intensity of the peak on the compression side of the (110) plane increases at *τ* = 50 ps. As the intensity increases with time, it transforms into a new peak, which is the Bragg peak for the shock-compressed bcc (110) plane. This is the main target of our analysis.

Figure 2g shows one dimensional profiles for a series of delay times. The intensity of the new peak, which is significantly compressed compared to the initial peak, is low at *τ* = 50 ps, but it increases significantly from *τ* = 150 ps to 700 ps and decreases after *τ* = 800 ps.

**Figure 3:**
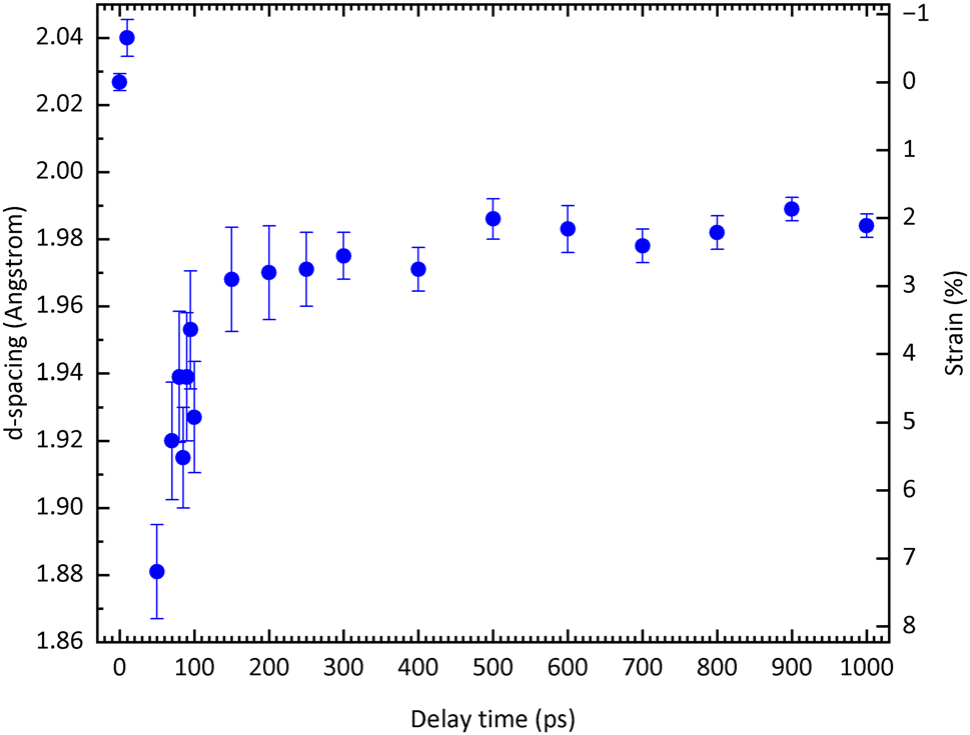
Temporal evolution of the lattice spacing *d* and the corresponding elastic strain *ε*^e^ for a shock-compressed bcc (110) plane. Lattice spacing *d* (blue dots) and the corresponding compressive elastic strain *ε*^e^ are shown. Error bars for the lattice spacing indicate the full width at half maximum (FWHM) of the Bragg peaks for the shock-compressed bcc (110) plane. The d-spacing increases at *τ* = 10 ps, presumably due to lattice expansion caused by the rapid energy transfer from the electrons in the higher energy state due to inverse bremsstrahlung to the lattice. The lattice spacing decreases drastically to 1.88 Å at *τ* = 50 ps, corresponding to an elastic strain *ε*^e^ of 7.19% at a compressive strain rate of 1.96 × 10^9^ s^−1^. After *τ* = 50 ps, the lattice spacing starts to expand. The expansion rate is initially high (− 4.05 × 10^8^ s^−1^ for 50 ps ≤ *τ* ≤ 150 ps), while it becomes moderate after 150 ps (− 9.29 × 10^6^ s^−1^ for 150 ps ≤ *τ*  ≤ 1 ns). The FWHM, which reflects the plasticity, increases between *τ* = 50 ps and 150 ps and then gradually decreases.

Figure 3 shows the lattice spacing *d* of the shock-compressed bcc (110) plane and the corresponding nominal compressive elastic strain *ε*^e^ = (*d*_0_ – *d*)/*d*_0_ as a function of the delay time *τ*, where *d*_0_ is the initial lattice spacing of 2.0268 Å. The error bar shows the full width at half maximum (FWHM) in the fitting profile. Note that the direction normal to the lattice plane is nearly parallel to the direction of shock propagation normal to the surface. The lattice spacing in the shocked region decreases drastically to 1.88 Å at *τ* = 50 ps, corresponding to a compressive elastic strain *ε*^e^ of 7.19%. At *τ* = 10 ps, the peak from the shocked region is absent and lattice expansion is confirmed. Therefore, the compressive elastic strain rate ${\dot{\varepsilon}}^\text{e}$ from the initial state to 50 ps is 1.96 × 10^9^ s^−1^.

After *τ* = 50 ps, the lattice spacing in the shocked region begins to expand. The expansion rate is initially high, but becomes moderate after *τ* = 150 ps. The corresponding elastic strain rate ${\dot{\varepsilon}}^\text{e}$ is − 4.05 × 10^8^ s^−1^ from *τ* = 50 ps to 150 ps and − 9.29 × 10^6^ s^−1^ after *τ* = 150 ps. The peak width increases from *τ* = 50 ps to 150 ps, and then it gradually decreases.

**Figure 4:**
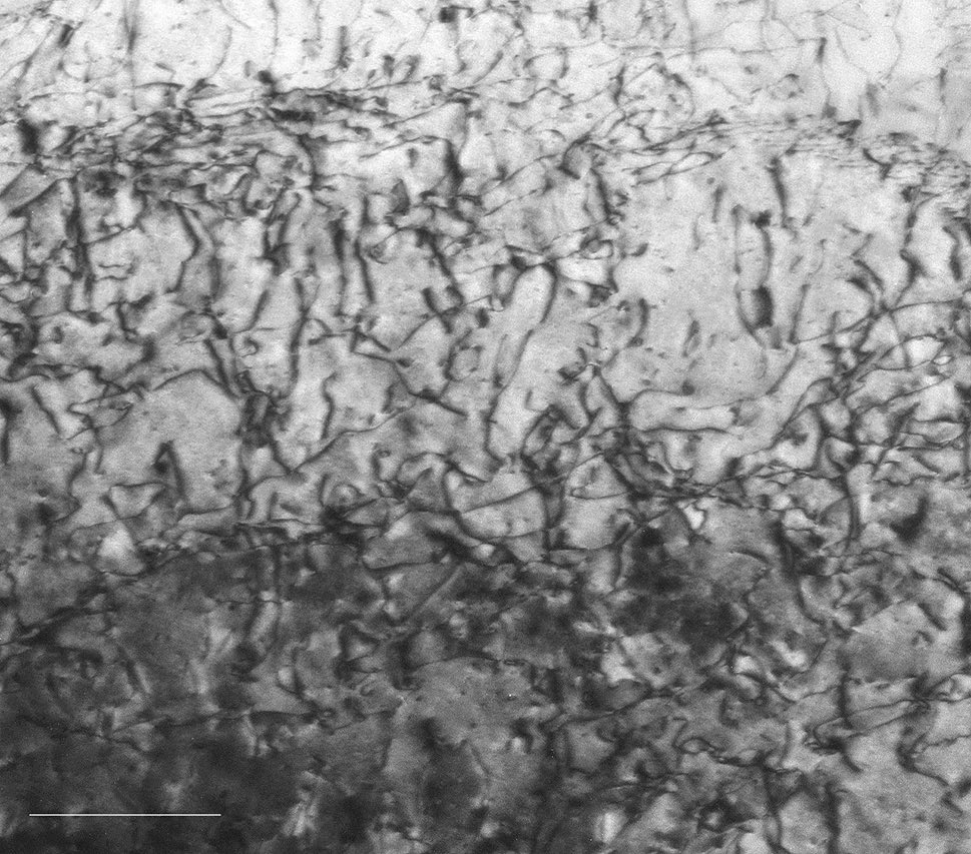
Transmission electron microscope image of shock-compressed iron within the probe depth of the XFEL pulse. The scale bar is 200 nm in length. This image shows high density dislocations, which is the trace of plastic deformation. The estimated dislocation density is of the order of 10^15^ m^−2^, while the initial density is of the order of 10^12^ m^−2^.

Figure 4 shows the TEM image of shock-compressed iron within the probe depth of the XFEL pulse. This image shows high-density dislocations of the order of 10^15^ m^−2^ while the initial density is of the order of 10^12^ m^−2^, indicating severe plastic deformation is taking place.

## Discussion

The slight increase in the d-spacing of the bcc (110) plane at *τ* = 10 ps is considered to be due to lattice expansion caused by the rapid energy transfer from the electrons in the higher energy state due to inverse bremsstrahlung to the lattice^24,40^.

The stress under uniaxial elastic compression is expressed as *σ*_*x*_ = (*C*_11_ + *C*_12_ + 2*C*_44_)* ε*_*x*_^e^/2, where *x* is the compression direction, *C*_11_, *C*_12_ and *C*_44_ are the elastic stiffness and *ε*_*x*_^e^ is the elastic strain along the compression direction. Assuming that the drastic change at *τ* = 50 ps is due to uniaxial elastic compression as in conventional shock compression, using *C*_11_ = 233.1 GPa, *C*_12_ = 135.44 GPa and *C*_44_ = 117.83 GPa at 300 K^55^ and the compressive elastic strain *ε*^e^ of 7.19% at *τ* = 50 ps, the stress *σ* loaded normal to the bcc (110) plane is 21.7 GPa. This is almost two orders of magnitude higher than the yield strength under static compression^56^. To confirm the validity of the uniaxial elastic compression assumption, this value of *σ* was compared with the yield stress obtained experimentally under high strain rate deformation^47^. For iron, the peak elastic precursor stress *σ*_E_, which corresponds to the stress at the onset of plastic deformation, was measured up to the strain rate at the onset of plastic flow $\dot{\varepsilon }$ of 10^8^ s^−1^ to obtain the relationship *σ*_E_ – *βd*^–1/2^ = 2.3 × 10^–3^ $\dot{\varepsilon }$ ^0.43^, where *β* = 0.69 and *d* is the grain size^47^. The value of *σ*_E_ = 23 GPa was obtained by extrapolating the strain rate $\dot{\varepsilon }$ of 1.96 × 10^9^ s^−1^ from *τ* = 10 ps to 50 ps obtained in this experiment and substituting *d* = 63 µm used in this experiment. These two values were in good agreement, indicating the suitability of the initial assumption that the drastic change at *τ* = 50 ps is due to uniaxial elastic compression in the same way as conventional shock compression.

Under conventional shock compression, the bcc to hcp phase transition of iron begins at 13 GPa and ends at 20 GPa^42–53^. The d-spacing of the (101) plane of the hcp structure in the Hugoniot state is 1.8825 Å at 13 GPa and 1.8648 Å at 20 GPa and that of the (100) plane is 2.1405 Å at 13 GPa and 2.1205 Å at 20 GPa. These peaks are not confirmed in Fig. 2a′ to f′, indicating that the femtosecond laser-driven shock wave does not induce the high pressure phase with the hcp structure at this time scale. The d-spacing of the bcc (110) plane is 1.9828 Å at 13 GPa and 1.9643 Å at 20 GPa^45^. These values agree well with the d-spacing values obtained in this experiment at *τ* = 1 ns and 150 ps, respectively. The decrease in elastic expansion strain rate and plasticity of the bcc structure after 150 ps may be influenced by the bcc to hcp phase transition. The sluggish phase transition of iron from bcc to hcp structure, despite its non-diffusive nature, remains an unresolved problem. The relaxation time of this transition depends on the shock pressure, i.e. with longer relaxation time at lower shock pressure, e.g. approximately 60 ns to 12 ns for the shock pressure of 17 GPa to 30 GPa^44^. As the peak stress measured in this experiment was 21.7 GPa, the shock wave required a relaxation time of at least 12 ns to complete the bcc to hcp phase transition. Therefore, no peaks of the hcp structure were observed during this measurement but could be observed later.

The width of the XRD peak qualitatively reflects the amount of plasticity or the number of lattice defects such as dislocations. The behaviour of the peak width, which increases from *τ* = 50 ps to 150 ps and then gradually decreases, is consistent with the observed plasticity trend, which also increases from *τ* = 50 ps to 150 ps and then gradually decreases. Therefore, the lattice behaviour shown in Fig. 3 can be interpreted as follows. At *τ* = 50 ps, a significantly large uniaxial elastic compression and many lattice defects are introduced, followed by a rapid elastic expansion up to *τ* = 150 ps and a gradual elastic expansion after *τ* = 150 ps with a decrease in the number of lattice defects. This behaviour is qualitatively consistent with an experimentally confirmed report that a shock-compressed material initially behaves as a purely elastic medium, eventually leading to plastic deformation^57–59^.

The shock pressure required for homogeneous nucleation of dislocations behind the shock front in iron is 8.6 GPa^7^. The peak elastic stress of 21.7 GPa for the femtosecond laser-driven shock wave estimated in this experiment was sufficiently higher than this value to allow the formation of the interface^60^ that allows a homogeneous nucleation of dislocations behind the femtosecond laser-driven shock front. The dislocation density at the interface at the shock pressure of 21.7 GPa was estimated to be 1.38 × 10^16^ m^−2^ (Supplementary Information). The elastic strain energy *μρb*^2^/2 of the dislocations was 3.3 × 10^7^ J/m^3^, where *μ* is the shear modulus and *b* is the Burgers vector. Here, the most elastically compressed state at *τ* = 50 ps and the state with the largest peak width at *τ* = 80 ps are compared. As the stresses at *τ* = 50 ps and 80 ps were 21.7 GPa and 18 GPa and the corresponding elastic strains were 7.19% and 5.29%, respectively, the difference in elastic strain energy *σε*/2 was 3.4 × 10^7^ J/m^3^, which agrees well with the value of the elastic energy of dislocations. The strain energy stored by the large elastic compression at *τ* = 50 ps was used to generate dislocations, which occurred from *τ* = 50 ps to 150 ps, resulting in the formation of the remaining high density dislocations observed in the directly femtosecond laser irradiated iron within the probe depth of the XFEL pulse, as shown in Fig. 4, where the dislocation density was estimated to be of the order of 10^15^ m^−2^, whereas the initial density was of the order of 10^12^ m^−2^.

The experimental results show that under femtosecond laser-driven shock compression the lattice was in a state of uniaxial elastic compression up to *τ* = 50 ps, an elasto-plastic hydrostatic compression or the Hugoniot state after *τ* = 150 ps, and an intermediate state between *τ* = 50 ps and 150 ps. This result is in good agreement with MD simulation results^28^, which show that when Cu was subjected to shock waves with rise times of 0 and 50 ps, it was initially in a state of uniaxial compression in the direction of shock wave propagation (one-dimensional compression), followed by a hydrostatic three-dimensional compression state to relax the uniaxial compression strain. Thus, the initial compression state caused by the shock wave driven by direct femtosecond laser irradiation is the same as that caused by conventional shock waves.

Based on these observations, we estimated the total strains in the compressed iron for 0 < *τ* ≤ 1 ns. The total strain *ε*^T^ is expressed as the sum of the elastic strain *ε*^e^ and the plastic strain *ε*^p^, i.e. *ε*^T^ = *ε*^e^ + *ε*^p^. In the initial region before *τ* = 50 ps under uniaxial elastic compression, *ε*^T^ = *ε*^e^. After *τ* = 150 ps, $\varepsilon^{{\text{T}}}$ is expressed as *ε*^T^ = 3*ε*^e^ due to the isotropic process (Supplementary Information). The stress at *τ* = 50 ps included only an elastic component of 21.7 GPa. As the material was in the Hugoniot state, the stresses of 19 GPa at *τ* = 150 ps and 14 GPa at *τ* = 1 ns were obtained after *τ* = 150 ps^45^.

**Figure 5:**
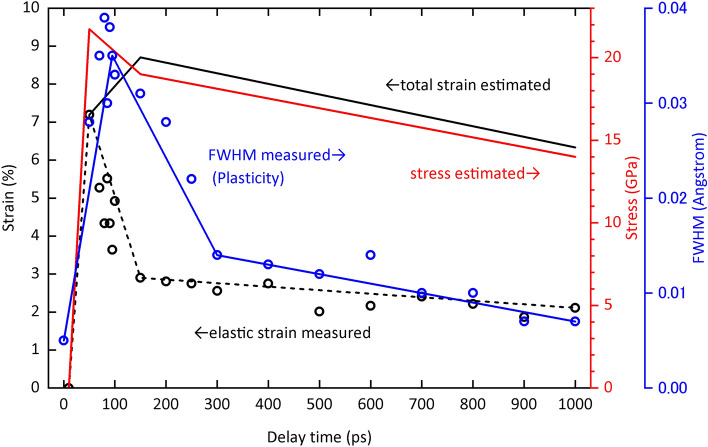
Temporal evolution of measured elastic strain (black circles and dashed line), estimated total strain (solid line), estimated stress (red line), and measured FWHM of peaks (blue circles and line). The stress wave peak precedes the plastic wave peak, as indicated by the peak width of the diffraction pattern, followed by the strain wave peak. Although it was theoretically predicted that the peaks of the stress and strain waves would diverge with time, this has not been reported experimentally. Furthermore, it is a novel finding that the plastic wave peak is positioned between these deviations, which has never been predicted even theoretically.

Figure 5 shows the measured elastic strain and FWHM of the diffraction peak, and the total strain and stress estimated from these measured data as a function of time, i.e. the temporal distribution of elastic, plastic, strain and stress waves, respectively. The time of maximum value of the temporal distribution of each wave is the earliest for stress and elastic waves, followed by plastic wave and then strain wave. It was theoretically predicted^61^ but shown here experimentally for the first time that the peaks of the stress and strain waves diverge with time, as significant dissipation and dispersion processes occur when the medium is subjected to compression or tension. Furthermore, the fact that the peak of the plastic wave lies between these stress and strain wave peaks is a new finding that has never been predicted theoretically. The findings of this study contribute to the understanding of the complex mechanisms of mechanics under shock compression, such as precursor decay anomaly and dynamic yielding, which have remained unanswered for the past 50 years^57–59^.

In summary, using XFEL diffraction measurements, we have successfully demonstrated the complex behaviour of stress, strain and plasticity in iron subjected to the shock wave driven by direct femtosecond laser irradiation. It is not possible to determine directly from the results of this study whether these behaviours are unique to the material subjected to the femtosecond laser-driven shock wave or can also be caused by the conventional shock wave. However, it is worth investigating further as such ultrafast behaviours caused by conventional shock waves have not been reported before. After *τ* = 150 ps, the material is in the Hugoniot state despite the expansion process under compression, which means that there may be other unknown waves hidden. Therefore, the femtosecond laser-driven shock wave is a suitable tool to probe the nature behind and possibly within the shock front. Furthermore, these features can persist in the material, allowing unique events such as DLP^17,18^ and high-pressure phase quenching^16^ that would not be possible with conventional shock waves. Further research into the lattice behaviour under femtosecond laser-driven shock compression will open up new avenues for future applications of femtosecond lasers as shock drivers.

### Supplementary Information

Supplementary Information.

## Data Availability

The datasets generated during and/or analysed during the current study are available from the corresponding author on reasonable request.
