# Supplementary material for: X-ray free electron laser observation of ultrafast lattice behaviour under femtosecond laser-driven shock compression in iron
Source: Sci Rep. 2023 Aug 31;13:13796. doi: 10.1038/s41598-023-40283-6 (PMC10471609; doi:10.1038/s41598-023-40283-6)
Supplement: Supplementary file 1 — Supplementary Information. [file 41598_2023_40283_MOESM1_ESM.docx]

**Supplementary Information**

**Total strain of plastically deformed material hydrostatically loaded under uniaxial strain condition**

Here we consider a cubic material whose *y*-*z* plane is shocked along the *x*-axis. We define the strain in the *x*-, *y*-, and *z*-axes as $\varepsilon_{x}$, $\varepsilon_{y}$, and $\varepsilon_{z}$, respectively. The total strain $\varepsilon^{\text{T}}$ is expressed as the sum of the elastic strain $\varepsilon^{\text{e}}$ and the plastic strain $\varepsilon^{\text{p}}$, i.e. $\varepsilon^{\text{T}}=\varepsilon^{\text{e}}+\varepsilon^{\text{p}}$. In the case of uniaxial strain, $\varepsilon_{y}^{\text{T}}$ and $\varepsilon_{z}^{\text{T}}$ are expressed by

$\varepsilon_{y}^{\text{T}}=\varepsilon_{y}^{\text{e}}+\varepsilon_{y}^{\text{p}}=0$, $\varepsilon_{z}^{\text{T}}=\varepsilon_{z}^{\text{e}}+\varepsilon_{z}^{\text{p}}=0$. (Seq. 1)

The following relationship applies in a state where the material is plastically deformed under constant volume;

$\varepsilon_{x}^{\text{p}}+\varepsilon_{y}^{\text{p}}+\varepsilon_{z}^{\text{p}}=0$. (Seq. 2)

Combining equation S1 with equation S2, the total strain in the *x*-axis of the material being plastically deformed under uniaxial strain condition is expressed by

$\varepsilon_{x}^{\text{T}}=\varepsilon_{x}^{\text{e}}+\varepsilon_{y}^{\text{e}}+\varepsilon_{z}^{\text{e}}$. (Seq. 3)

Furthermore, if the stress state is hydrostatic, i.e. $\varepsilon_{x}^{\text{e}}=\varepsilon_{y}^{\text{e}}=\varepsilon_{z}^{\text{e}}$, the total strain in the *x*-axis of the material is given by

$\varepsilon_{x}^{\text{T}}=3\varepsilon_{x}^{\text{e}}$. (Seq. 4)

**Dislocation density at Smith’s interface**

The shock pressure *P* as a function of the dislocation density *ρ* generated at the Smith's interface during shock compression is expressed by [S1]

$P=\frac{C_{0}^{2}\left\{1-\left\{1-\sqrt{2}\left[\frac{0.8b^{2}\left(1-\nu\right)\rho}{2\pi^{2}}\right]^{1/3}\right\}^{3}\right\}}{V_{0}\left\{1-S\left\{1-\sqrt{2}\left[\frac{0.8b^{2}\left(1-\nu\right)\rho}{2\pi^{2}}\right]^{1/3}\right\}^{3}\right\}^{2}}$ , (Seq. 5)

where *C*_0_ and *S* are the material specific equation of state parameters, *b* is the Burgers vector, *V*_0_ is the specific volume at ambient conditions, *ν* is equal to Poisson’s ratio. In case of iron, *C*_0_ = 3935 m/s, *ν* = 0.293, *b* = 0.2485 nm, *S* = 1.578, *V*_0_ = 1.270×10^-4^ m^3^/kg.

**Temperature Evaluation**

Temperature evaluation at intensities where ablation does not occur has long been carried out both theoretically and experimentally [S2-S6]. However, when metals are irradiated with femtosecond lasers of intensity exceeding the ablation threshold, as in the present study, ablation occurs with free electron acceleration and collisional ionisation, resulting in a very complex mixture of four states - solid, liquid, gas and plasma - and neither theoretical prediction nor experimental measurement of temperatures reflecting these four states has been achieved. Neither theoretical predictions nor experimental measurements of temperatures reflecting these four states have yet been achieved.

If this research progresses and the mechanical behaviour during femtosecond laser-driven shock compression is clarified, and the time of the Hugoniot state is known, it will be possible to estimate the temperature from the stresses obtained from the experiments using thermodynamics based on the Hugoniot state after that time [S7-S9]. For example, in the present study, it has already been shown that the temperatures at τ = 150 ps and τ = 1 ns would be 408 K and 360 K, respectively [S10-S12], if they existed on the Hugoniot state, since d-vals measured at τ = 150 ps and τ = 1 ns correspond to d-vals of 20 GPa and 13 GPa of the bcc structure on the Hugoniot state, respectively.

During the time in thermodynamic non-equilibrium from immediately after femtosecond laser irradiation to the Hugoniot state, it is extremely difficult to estimate the temperature (energy state) theoretically, and its actual measurement is necessary. For example, in-situ XAFS and absorption spectroscopy measurements using XFEL are currently planned [S13], and these are at the forefront of modern scientific research.

**The positional relationship between the femtosecond laser irradiated area and the XFEL detecting area**

The d-value at *τ* = 150 ps is the same as the d-value of the bcc structure at 20 GPa on the Hugoniot; since the shock velocity at 20 GPa is about 5 km/s, this shock wave penetrates the XFEL skin depth at about 200 ps. Therefore, the region where the scattered XFEL signal is acquired has three phases in the depth direction: Laser-heated area, Shock-compressed area and the unaffected region up to around *τ* = 200 ps, and two phases, Laser-heated area and Shock-compressed area, after *τ* = 200 ps (Fig. S1). Actually, the time when only these two phases present is earlier than 200 ps, as stronger (faster) shock waves are expected to be present between *τ* = 10 ps and 50 ps. The shock velocity is sufficiently larger than the heat front velocity to provide a pure shock-compressed area deeper than the heat front. The new peak targeted in this paper, shown in Fig. 2, is a signal from this pure shock-compressed area deeper than the heat front.

The reasons for the presence of the uncompressed BCC(110) peak even after 200 ps, when two phases, Laser-heated area and Shock-compressed area, are present in the depth direction, are described. The femtosecond laser used in this experiment has a near Gaussian spatial intensity profile of M^2^ ~ 1.5. Therefore, the shock wave is driven in the intensity region above the shock-driving threshold, while the shock wave is not driven in the region below it, and only the laser heat effect is given to the material. The positional relationship between the femtosecond laser irradiated area and the XFEL detecting area is shown in Fig. S2. The XFEL detecting area consists of a shock compressed area and a laser heated area. In Fig. 2 a'-f'&. g, the peaks with d-values close to the initial value of 2.026 A of BCC(110) are signals from the laser-heated area, while the new peaks with smaller d-values, which are the target of this paper, are signals from the shock-compressed area.

The femtosecond laser used in this experiment has a near Gaussian spatial intensity profile of M^2^ ~ 1.5. Therefore, the shock wave is driven in the intensity region above the shock-driving threshold, while the shock wave is not driven in the region below it, and only the laser heat effect is given to the material. The positional relationship between the femtosecond laser irradiated area and the XFEL detecting area is shown in Fig. S1. The XFEL detecting area consists of a shock compressed area and a laser heated area. In Fig. 2 a'-f' & g, the peaks with d-values close to the initial value of 2.026 A of BCC(110) are signals from the laser-heated area, while the new peaks with smaller d-values, which are the target of this paper, are signals from the shock-compressed area.


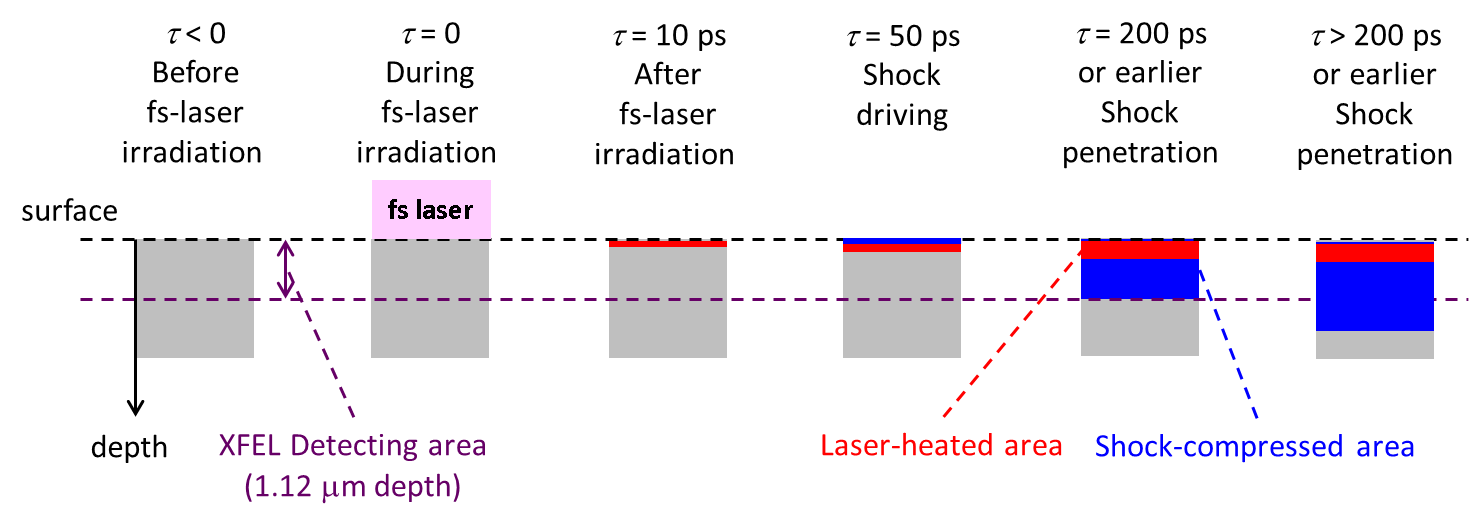


Fig. S1. Schematic illustration of laser-heated area and shock-compressed area in depth.


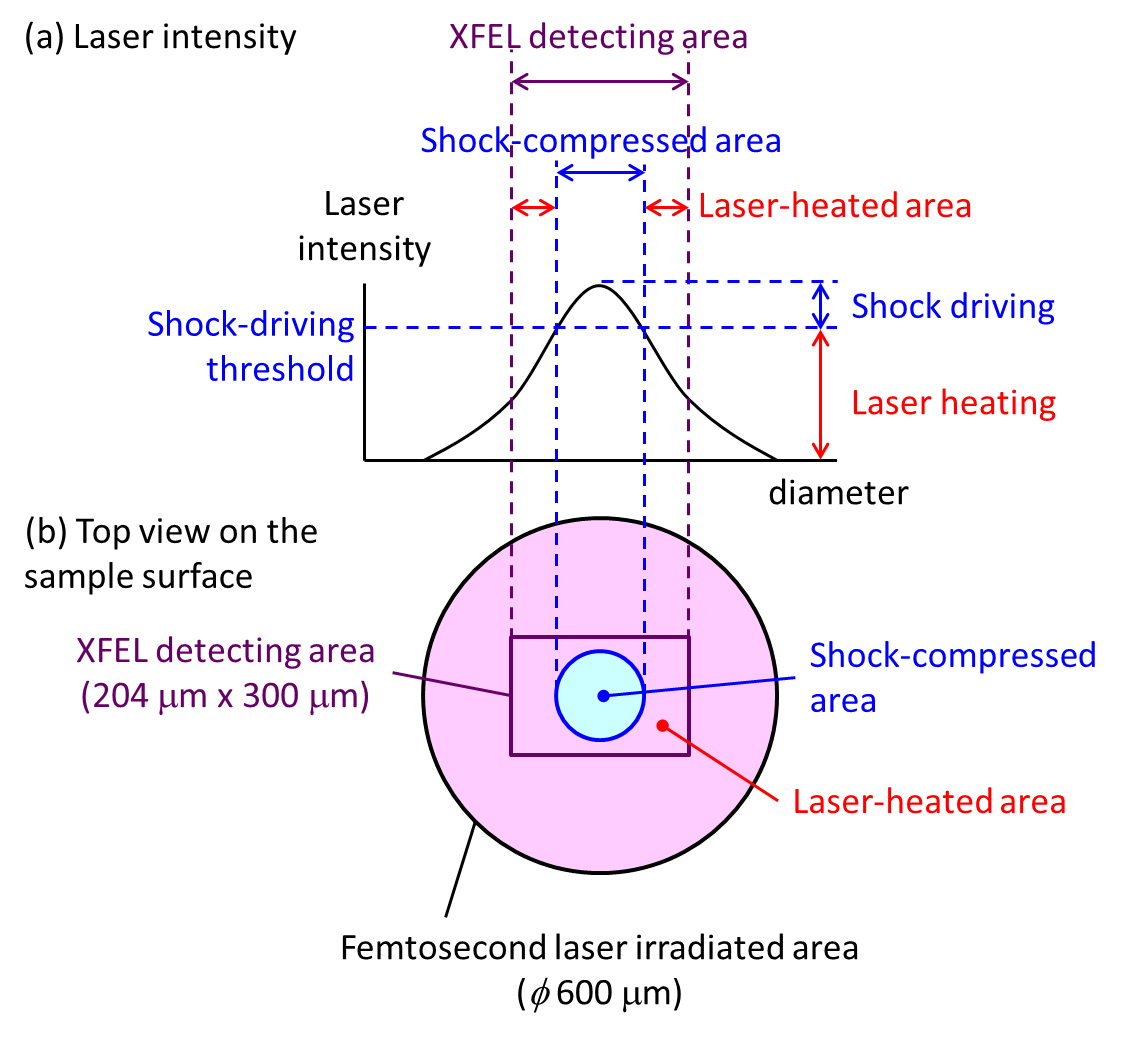


Fig. S2. XFEL detecting area consists of shock-compressed area and laser-heated area.

**References for supplementary information**

1. M.A. Meyers, F. Gregori, B.K. Kad, M.S. Schneider, D.H. Kalantar, B.A. Remington, G. Ravichandran, T. Boehly, J.S. Wark, Acta Mater. 51 (2003) 1211.
2. Kaganov et al., Sov. Phys. JETP 4, 173 (1957).
3. Anisimov et al., Sov. Phys. JETP 39, 375 (1974).
4. G.L. Eesley, Phys. Rev. B 33, 2144 (1986).
5. Corkum et al., Phys. Rev. Lett. 61, 2886 (1988).
6. Chichkov et al., Appl. Phys. A 63, 109 (1996).
7. Walsh and Christian, Phys. Rev. 97, 1544 (1955).
8. Wallace, Phys. Rev. B 22, 1477 (1980).
9. Wallace, Phys. Rev. B 24, 5597 (1981).
10. Boettger and Wallace, Phys. Rev. B 55, 2840 (1997).
11. Sano and Sano, J. Appl. Phys. 90, 3754 (2001).
12. Sano and Sano, J. Appl. Phys. 90, 5576 (2001).
13. 23rd Biennial Conference of the APS Topical Group on Shock Compression of Condensed Matter, June 19–23, 2023; Chicago, Illinois, USA
